# Whose Responsibility is Adolescent’s Mental Health in the UK? Perspectives of Key Stakeholders

**DOI:** 10.1007/s12310-018-9263-6

**Published:** 2018-04-06

**Authors:** Michelle O’Reilly, Sarah Adams, Natasha Whiteman, Jason Hughes, Paul Reilly, Nisha Dogra

**Affiliations:** 1 The Greenwood Institute of Child Health, University of Leicester, Westcotes Drive, Leicester, LE3 0QU UK; 2 University of Leicester, University Road, Leicester, LE1 7RH UK; 3 Information School, University of Sheffield, 235 Regent Court, 211 Portobello, Sheffield, S1 4DP UK

**Keywords:** Mental health, Adolescents, Responsibility, Schools, Qualitative

## Abstract

The mental health of adolescents is a salient contemporary issue attracting the attention of policy makers in the UK and other countries. It is important that the roles and responsibilities of agencies are clearly established, particularly those positioned at the forefront of implementing change. Arguably, this will be more effective if those agencies are actively engaged in the development of relevant policy. An exploratory study was conducted with 10 focus groups including 54 adolescents, 8 mental health practitioners and 16 educational professionals. Thematic analysis revealed four themes: (1) mental health promotion and prevention is not perceived to be a primary role of a teacher; (2) teachers have limited skills to manage complex mental health difficulties; (3) adolescents rely on teachers for mental health support and education about mental health; and (4) the responsibility of parents for their children’s mental health. The research endorses the perspective that teachers can support and begin to tackle mental well-being in adolescents. However, it also recognises that mental health difficulties can be complex, requiring adequate funding and support beyond school. Without this support in place, teachers are vulnerable and can feel unsupported, lacking in skills and resources which in turn may present a threat to their own mental well-being.

## Introduction

Mental health difficulties have been increasingly framed as conditions of the young (Howard, Burton, Levermore, & Barrell, 2017). In the United Kingdom (UK), concerns were raised about the impact of mental health difficulties on adolescents’ educational and work prospects, physical health and mortality [Department of Health (DoH) & National Health Service England (NHS), 2015]. The risk that deteriorations in mental health may continue through into adulthood [Chief Medical Officer (CMO), 2012] has also raised concern, especially since an estimated 50% of adults with mental health difficulties reported they first experienced these before the age of 15 years (Kessler, Berglund, Demler, Jin, & Walters, 2005). Accordingly, it has been suggested that preventative action, early intervention and recovery utilising a multi-agency and multidimensional approach should be put in place to support adolescents (Department of Health (DoH), 2015). Such multidimensional responses recognise that the aetiology of mental health difficulties involves a complex nexus of relationships between society, family and school that can generate, as well as ameliorate, the problems adolescents experience (Weare, 2000).

In the UK, data suggested that 10% of 5–15-year olds had a diagnosable mental disorder, or roughly one in three children/adolescents in an average classroom (Green, McGinnity, Meltzer, Ford, & Goodman, 2005). International surveys have reported similar results, with an estimated 10–20% of children and adolescents worldwide said to have experienced these difficulties (Keiling et al., 2011). More recently, a survey conducted by The Key^1^ (2017) found that school leaders reported a recent increase in mental health difficulties in adolescents; and secondary (high) school leaders reported an 87% increase in stress, anxiety and panic attacks, an 80% increase in depression, and a 75% increase in incidences of self-harm compared to 2 years previously. This is consistent with work produced by the Royal College of Paediatrics and Child Health (RCPCH, 2017) which showed an increase in incidents of self-harm in children and adolescents. Further evidence of this increasing prevalence can be found in a comparison of a study by Wood (2009) which reported a 10% prevalence rate of self-harm with a study 5 years later by the World Health Organisation (WHO, 2014) that demonstrated a prevalence rate of 20%. However, these findings and the increase they point towards may in part have been influenced by campaigns to increase awareness and improved identification of difficulties.

The concerns outlined above have prompted the publication of numerous guidelines on how to support children and adolescents with mental health difficulties from within the education and health sectors [Department for Education (DfE), 2016; DoH & NHS England, 2015; House of Commons Education and Health Committees (HCEHC), 2017]. The education system was positioned as well-placed to support the development of positive mental health and well-being due to their daily contact with adolescents during school hours (HCEHC, 2017). Teachers are acknowledged as being key adults in an adolescent’s life who may first see the signs of emerging difficulties (DfE, 2016). Additionally, schools, as stated in the Education Act (2002) and Academies Act (2010), are responsible for supporting the mental development of students (DfE, 2014) while also providing for those identified as having Social, Emotional and Mental Health (SEMH) needs. SEMH includes those that may have an underlying mental health difficulty, i.e., emotional, behavioural or neurodevelopmental (DfE, 2015, p. 97)

Arguably, for teachers to recognise mental health difficulties in adolescents and provide appropriate support, they need to have been adequately trained in this area and feel competent to conduct that role. However, research has suggested that mental health difficulties are an area that teachers often feel least confident dealing with (Rothi, Leavey, & Best, 2008), lacking skills and training (Reinke, Stormont, Herman, Puri, & Goel, 2011), and not always being able to differentiate between typical and atypical mental health (Loades & Mastroyannopoulou, 2010). To address these concerns, the UK government has recently pledged that all secondary (high) schools in England will receive mental health training by 2020 [Public Health England (PHE), 2017]. While this is a positive move to upskill those working within education, it perhaps underestimates factors which could impede its success. There is also a misguided assumption that quality training in mental health is easily provided and immediately translates into effective practical implementation. Kidger, Gunnell, Biddle, Campbell and Donovan (2009) suggested that teachers themselves may be hindered in their ability to support adolescents with mental health difficulties due to experiencing their own conditions. Stressors within teaching that may impact on teachers’ mental health included: excessive workloads, adolescent’s behaviour, school targets, examinations and inspections (Naghieh, Montgomery, Bonell, Thompson, & Aber, 2015).

Such issues are compounded by an education system that appears to put greater emphasis on academic achievement than on the personal and social well-being of adolescents (HCEHC, 2017). While well-being is recognised as important, Personal, Social, Health and Economic Education^2^ (PSHE) remains a non-statutory subject (DfE, 2014), which arguably limits the promotion of positive mental health across the entire curriculum. Nonetheless, reports have suggested that where schools have procedures in place to support children’s well-being, attainment levels are higher (DfE, 2014). Accordingly, efforts to encourage academic attainment and positive mental health demonstrate the two can work in harmony rather than in opposition (PHE, 2014). Yet to date, there has been a lack of evidence-based research relating to successful approaches to mental health and well-being within schools.

One approach which has been identified as being particularly successful is the Targeted Approach to Mental Health in School, which requires a ‘whole school approach’ (Weare, 2000). The DoH and DfE (2017) suggest that a whole school approach to mental health and well-being should be led by a designated senior leader and be reflected in a school’s behaviour policy, curriculum design, care and support of adolescents and staff, with parental engagement. In this approach, there should be clear and consistent implementation of targeted approaches to promote positive mental health throughout the school drawing upon evidence-informed practice. A challenge to a whole school approach is ensuring all staff commit. For example, in their research Kidger et al. (2009) highlighted variances in teachers’ stances on whether their role was to cater for a child’s well-being alongside their academic obligations.

Importantly, schools should be viewed as only one part of a multi-agency approach towards the promotion of adolescent mental health and well-being (PHE, 2015). Adolescents with complex mental health needs may find it hard to trust their teachers and may not wish to highlight their issues to their peers by drawing attention to them in the school environment (Hart & O’Reilly, 2017). This may especially be the case when school factors may in part be related to the mental disorder. It is also important not to overestimate what educators can do in meeting existing complex mental health needs. As Williams and Salmon (2002) suggested, adolescents with complex problems require multidisciplinary input and specialist services, and teachers will not have the necessary skills and knowledge to meet them without the support of specialist staff. In the UK, Child and Adolescent Mental Health Services (CAHMS) usually operate as multidisciplinary teams who work with other agencies as required. Within this model CAHMS specialists may work jointly with professionals in education and social care to provide a collaborative approach to meeting the child’s needs. Indeed, the demand for such specialist input is rising. CMO (2012) highlighted that there has been an increased demand for the service particularly for adolescents with more complex and severe problems. However, access and funding throughout England varies with the spend per head ranging from £2.01 ($2.70) to £135.85 ($183.56) [Royal College of Psychiatrists (RCOP), 2016], reflecting a more general inadequacy of supporting mental health economically [London School of Economics and Political Science (LSEPS), 2012]. Notably, as Docherty and Thornicroft (2015) have reported, there has been a substantial reduction in the resources targeted at supporting mental health care, which makes improving the mental health outcomes for adolescents a challenge.

Caring for children including attending to their mental health is clearly a complex issue that requires the appropriate input of an array of stakeholders including teachers and other educationalists, health practitioners, adolescents and their families. With the increased pressure on schools to prevent mental illness and promote mental well-being it is important to understand the perspectives of those most affected. This paper aims to explore, from the perspective of educational professionals, mental health practitioners and adolescents who should be taking responsibility for adolescents’ mental health and the practical implications of these suggestions made by the various stakeholders. The understanding of educational professionals, mental health practitioners and adolescents is important since an increased responsibility and pressure on school personnel has not been synonymous with an increased capacity to deal with adolescent mental health difficulties. While there has been a greater load of political expectation placed upon education in this respect, there has been limited work exploring the views of those on the frontline. For this paper, we therefore ask; ‘whose responsibility is adolescent mental health and mental illness?’ which iteratively developed post data collection from a broader research question about mental health promotion.

## Methods

The exploratory character of the research question promoted a qualitative design. Adolescent mental health in schools is a concern for public services and political advocates, and to contextualise this it is necessary to ensure that the perspectives of those of those working at the frontline in education and health are part of the evidence generated within this area.

### Focus Groups and Sampling

Participants were recruited in 2016/2017 from two large cities in the UK, Leicester and London and conducted separately in those cities. These areas represent the education system relatively well, with the London school being a large inner-city institution and the Leicester one being more suburban. This captures a large spread of age, gender, ethnicity and socio-economic status (but did not capture small rural areas).

Focus groups are well known to be an effective data collection method for health research (Wilkinson, Joffe, & Yardley, 2004) and were utilised due to their potential to encourage participants to share ideas and comment on the contributions of others (Willig, 2008). Initial contact was made with head teachers (Principals) and clinical managers via email. This was followed up by telephone calls and an official invitation to participate in the study. Head teachers coordinated recruitment of educationalists and adolescents, clinical managers facilitated recruitment of mental health practitioners. In total, 10 focus groups were conducted; six with adolescents aged 11–18 years (*N* = 54); two with mental health practitioners (*N* = 8) and two with educational professionals (*N* = 16), lasting between 45 min and 1-h, 15 min. Adolescent groups consisted of 30 males and 24 females, predominantly from White British and South Asian ethnicity; see Tables 1 and 2 for demographic detail.

**Table 1:** Demographics of the adolescents by gender

	Male	Female	Total
London	18	9	27
Leicester	12	15	27
Total	30	24

**Table 2:** Demographics of the adolescents by ethnicity

	White British	South Asian	African	Middle East	Eastern European	Other	Total
London	0	11	4	5	4	3	27
Leicester	15	7	0	4	1	0	27
Total	15	18	4	9	5	3	54

Our primary focus is on the perspectives of adult professionals, but this is supported in places by the voices of adolescents. The educationalist group consisted predominantly of teachers, but also included teaching assistants and head teachers. This group was aged from 23 to 59 years, and included six males and ten females. Eleven of the educationalists were White British, four South Asian and one Eastern European. The mental health practitioners consisted of two males and four females, aged 30–54 years, and two of those were White British and four South Asian (see Tables 3, 4).

**Table 3:** Demographics of professionals by gender

	Male	Female	Total
Educationalists	6	10	16
CAMHS	2	6	8
Total	8	16

**Table 4:** Demographics of professionals by ethnicity

	White British	South Asian	Eastern European	Total
Educationalists	11	4	1	16
CAMHS	2	6	0	8
Total	13	10	1	

Sampling adequacy was ensured by reaching data saturation, whereby no new ideas emerged across groups. This is congruent with a thematic approach (O’Reilly & Parker, 2013) and was assured both across the number of focus groups as well as participation within them (Hancock, Amankwaa, Revell, & Mueller, 2016). Saturation within and across groups was determined by the stopping criterion when no new issues were discussed (Francis et al., 2010) and was verified through the multiple-coder coding process.

All three groups were asked a general range of questions which were semi-structured in style to allow for participant-driven discussions to some extent. Participants were reminded of the core ethical principles and advised that the focus of the discussions was on social media (not discussed in this paper), mental health and promotion of well-being. Participants were advised of the ‘rules’ of conduct and confidentiality and were guided by the facilitator to share opinions, experiences and stories, who kept discussions on track and facilitated the participatory techniques in the adolescent groups. The schedule of questions was organised round three core categories that reflected the focus of the broader project, and included conceptualisations of mental health, opinions and experiences of social media, and mental health promotion. The issues of responsibility and schools came up predominantly in talking about mental health and risks to well-being and this was consistent across groups, despite not having specific questions in this area. Thus, while we did not ask questions about responsibility, all groups raised this when talking about mental health.

### Analytic Approach

A thematic approach was used because of its data-driven strategy and focus on meanings generated from participant perspectives (Braun & Clarke, 2006). This technique provided a mechanism for identifying the salient issues at stake for participants (Boyatzis, 1998). Data were transcribed verbatim and a manual coding frame created by multiple members of the team. In practice, this was a multi-layered approach, identifying first, second- and third-order codes (Boyatzis, 1998) by three team members. This provided a systematic technique for categorising the issues of relevance to all participant groups, and the three coding frameworks were mapped onto one another with agreement for labels being ascertained via meetings between coders. The broad project identified 122 second order codes, which were the main categories reflecting the areas of concern for participants (Boyatzis, 1998). These collapsed into ten superordinate themes, and their related themes under each rubric. The overarching theme of mental health responsibility is discussed in this paper, and the four themes within this superordinate section identified and discussed.

### Ethics

This study was given ethical approval from the University of Leicester Ethics Committee. The core ethical principles were carefully adhered to, and consent from all parties (and parents where required) was obtained. Anonymity was assured in the use of quotations.

## Findings

Participants provided a range of different views both about how schools support adolescents with mental health difficulties and how they educate them more generally about mental health: considerations that were often conflated by the participants. Notably, all three groups were generally consistent in types of issues they raised about responsibility, and thus, the quotations included here serve as reflective examples of each group and each of the main issues raised. Four key themes were identified under the rubric of mental health responsibility: (1) mental health promotion and prevention is not the primary role of a teacher; (2) teachers have limited skills to manage complex mental health difficulties; (3) adolescents rely on teachers for mental health support and education about mental health; and (4) the responsibility of parents for their children’s mental health.

### Theme One: Mental Health is Not the Primary Role of a Teacher

The most commonly recognised responsibilities of a teacher are to educate students and prepare them for key examinations. Teachers provide students with constructive feedback and academic support to promote academic progress and attainment. Adolescents, however, bring to the school environment more than just their learning needs. Accordingly, pastoral care (i.e., providing emotional support and promoting well-being) has found a necessary place on the education agenda. With the increasing prevalence of mental disorders, and the broader issues of preventing mental illness, adolescents are frequently turning to teachers and other educational staff for support with their emotional well-being especially as it can significantly impact on their learning. Subsequently, teachers are frequently finding themselves dealing with issues that they sometimes feel ill-equipped to manage. During the discussions, participants differentiated between supporting emotional well-being and mental health promotion, from managing existing diagnosed mental disorders and working with mental health services.

We’re not trained, it’s not our primary role because we’re here as getting them an education.

P1–London education

Well I think his best place is to be in the school but if he’s not mentally stable or ready to take on the challenges of school, then we are not the right place for it.

P4–Leicester education

It’s not our job to cure it [mental disorder].

P5–Leicester education

In discussions about those diagnosed with specific disorders, the educational professionals were clear that managing the behaviours and emotions related to those conditions was not part of their job. Indeed, they felt that schools were ‘not the right place for it’. Teachers and other educational professionals expressed the perspective that they were concerned about the increasing pressures being placed on schools to manage mental health difficulties, when their ‘primary role’ was to give these adolescents ‘an education’. The connotations of the verb ‘to educate’ suggest a tighter delineation to a teacher’s role—to be first and foremost engaged with imparting knowledge, perhaps fostering intellectual development, and only secondarily attending to supporting the emotional needs of different students.

Participants noted that to promote emotional well-being, assure mental health promotion and manage mental disorders within schools, teachers need appropriate resources to do so. They indicated that there was generally a lack of quality training and financial resources for them to make this part of their daily activities and they felt that mental health generally was badly funded.

We say we take children with mental health seriously but actually we fund it appallingly as a society.

P1–CAMHS1

Unfortunately, part of it does come down to resources.

P4–Leicester education

I was in a discussion with people who would be involved in the funding to hear the quote ‘children don’t kill themselves’. They do. Um, and to think that that attitude still, it is really painful to think about and we don’t want to think about children in our society killing themselves but they do.

P2–CAMHS 1

We’re not putting the resources in and, you know …

P7–Leicester education

The lack of funding for mental health was discussed in relation to educational resources and the poor funding for specialist services, which was an area of concern for participants. They noted that despite the rhetoric of mental health being a priority, ‘we actually fund it appallingly as a society’, commissioners of services are failing to recognise the reality that in severe cases children do commit suicide because of their distress ‘but they do’. Equally, in educational settings managing adolescents’ well-being ‘does come down to resources’ as ‘we’re not putting the resources in’. Of course, schools in the UK have been consistently affected by government austerity measures (Sims-Schouten, 2017), and arguably, they are insufficiently resourced to manage mental health difficulties as well as educational attainment. Furthermore, dealing with these issues is a time-consuming process. Teachers in our study repeatedly argued that they simply did not have sufficient time to manage these complex issues in addition to everything else they needed to do.

Today at lunchtime I had a group of students that I had to deal with and I had at the end I only had fifteen minutes to eat my lunch and to go over to my next classroom. I just don’t think we have enough time.

P3–London education

### Theme Two: Teachers Have Limited Skills to Manage Complex Mental Health Difficulties

Participants argued that teachers are not experts in mental health and are not trained sufficiently to impart knowledge about emotional well-being. Furthermore, it was recognised that teachers were not well-equipped to treat adolescents’ mental disorder symptoms and behaviours in the school environment.

I mean we’re not medically trained, that’s not our side.

P2–Leicester education

It seems to be more and more expected that school staff are supposed to be doing this yet there’s no time, there’s no training, there’s no supervision.

P1–London education

All we can do is signpost.

P8-Leicester education

Managing mental disorders is a specialist area requiring the expertise of a mental health practitioner in diagnosing and treating complex needs. Teachers, however, have limited training in mental health. While teachers may have some level of training, they are typically not ‘medically trained’^3^ and all they are able to do is ‘signpost’. Nonetheless, participants in the study alluded to the growing expectations of school staff to deal with such concerns, with a concomitant failure at the level of policy and governance to recognise the lack of ‘time’, ‘training’ or ‘supervision’ necessary for teachers properly to embrace this role. Interestingly, some participants did note that teachers were given training in some areas of mental health, but questioned the quality or impact of this.

And I think the lack of training of front line professionals and even though there has been quite a lot of training, I think some of it is non-contextual.

P1–CAMHS1

If we had a bit of training on how we would deal with it, like deal with these issues.

P4–London education

Although some training provided to teachers was reported to be useful in helping them deal with mental health ‘if we had a bit of training’, some participants questioned how helpful the training is if it is ‘non-contextual’. In other words, providing basic knowledge about specific disorders while de-contextualising from general areas of child development, was viewed as problematic. While basic mental health training may facilitate teachers to recognise problems and manage the growing problem, and may help with general mental health promotion, participants felt that this was insufficient in isolation to meet demand, because of time, resources and confidence.

I think there needs to be a separate unit dedicated to deal with mental health because I don’t feel like I’m doing it justice, I wish I could. I wish I could have more time, I wish I could be trained but you know

P3–London education

Due to this lack of training, participants felt that there was need for a ‘separate unit to deal with mental health’, one that reflected the necessary expertise to do it ‘justice’. This was constructed not as a lack of motivation on the part of teachers, but positioned as a point of necessity because of circumstances and the skills required. Importantly, the limitations of teachers to offer mental health support were also recognised by the young people themselves who felt that multiple sources of information were needed as teachers ‘might not have all of the knowledge about mental health’.

I think both because the teacher might not have all of the knowledge about mental health and the internet could help.

P4–London yr11

Notably, managing mental health in the school environment is not simply down to knowledge and training. Mental disorders carry a degree of risk, and mental health practitioners conduct a risk assessment as part of overall assessments (DoH, 2009). Indeed, some difficulties expressed by adolescents can be particularly risky, for example, self-harm, depression and substance misuse. Recognising and managing risk without training places education staff in danger of acting beyond their competency levels. Participants recognised the sensitivity of the issues they were dealing with and argued that education professionals often lacked the confidence to have mental health within their remit.

To know how to deal with a student if they came up to you and told you that they’ve got a mental illness or that they’re going through something. I’m not sure that I’d necessarily know what to say.

P1–Leicester education

So even if we had all the information in the world and we had sessions on different kind of mental health issues, but even if you have that, I still wouldn’t be confident talking to someone and giving them specific advice I don’t think.

P5–London education

In considering issues of confidence, educational professionals referred predominantly to working with adolescents with specific mental disorders, although there was also some discomfort with the idea of educating them about mental health in a broader sense. Participants felt that even with training in the area, they ‘wouldn’t feel confident’ in supporting those with mental disorders as they would not ‘know what to say’. Lacking the confidence to deal with children with diagnosed conditions in the school environment was also related to broader issues of secondary traumatic stress, as many working in education reported that managing this impacted significantly on their own mental well-being.

And we’ve had several suicide disclosures and the staff have lost their entire weekend worrying.

P1–London education

You take it on and you listen to what they’re saying but like you can’t like I try not to take it home and you try not to think about it, but you do because it’s, we care for these kids

P4–London education

Mental health practitioners typically have supervision within their roles to manage the risk of secondary traumatic stress or burnout. However, despite such supervision there is still a high level of stress and anxiety in the field as they deal with the traumatic narratives of others (DeVilly, Wright, & Varker, 2009). Those working in education are unlikely to have such support as dealing with mental health is not prerequisite of the educational role. Educationalists argued, however, that it was impossible not to be invested in the lives of their students. Consequently, those adolescents with mental health difficulties that posed some risk impacted heavily on teachers’ own mental health and well-being. When their students disclosed ‘suicide’, this resulted in those teachers ‘worrying all weekend’. Despite trying to compartmentalise these issues, teachers confessed that they were unable to just ignore the difficulties their students were encountering because ‘we care for these kids’. Therefore, the mental well-being of teachers is put at risk in the taking of responsibility for helping adolescents with such difficulties. In that sense, there was a general agreement that teachers spent time worrying about their students and also about one another.

I do worry about the staff, as well as the kids.

P1–London education

### Theme Three: Adolescents Rely on Teachers

It is important to recognise that participants moved fluidly between discussing supporting adolescents with diagnosed mental disorders and promoting mental well-being in a more general way. In discussions of mental health promotion and education in a general sense, there was some agreement amongst participants that teachers could help, although some educationalists still felt ill-prepared to do this fully. Nonetheless, participants did feel that talking openly about issues of mental health was important within the school environment.

We have a responsibility as a society and as an educational establishment to get the message across that it is okay to have these discussions.

P3–Leicester education

But I think ultimately, it’s the school’s problem.

P7–Leicester education

Notably, educationalists felt that discussing mental health issues was, at least in part, the responsibility of ‘an educational establishment’ in setting out to adolescents that it is ‘okay to have these discussions’ as ‘ultimately it is the school’s problem’. In other words, there was some agreement within the education focus groups that teachers could, and should, openly talk about mental health as part of their daily activity. This view was also shared by the young people themselves, who argued that being educated about mental health in school was important to them.

I think that’s mainly up to the school…..to do stuff like teach us about this

P6–London yr11

You don’t get taught about it at school like other things, like different illnesses or stuff. It’s just sort out, it’s just around school, like there’s people around that have it but you don’t know about it.

P5–Leicester yr11

Well, we have been taught it a bit but it’s not really been stressed as like a serious topic, like in real life. You don’t, you don’t really hear much about it ….. but quite a few people will just laugh it off and they don’t realise that it should be taught to young people.

P2–Leicester yr11

Adolescent participants often reported that there was a lack of open discussion about mental health in their schools, despite considering that it should be ‘taught to young people’. They suggested that there were opportunities within the school environments, such as in their PSHE or during school assemblies where teachers could provide them with information about mental health. They recognised that mental disorders were experienced by their peers, ‘there’s people around that have it’, but felt that they were uninformed about the effects ‘you don’t know about it’. Indeed, some argued that mental health is a ‘serious topic’, but they simply do not ‘hear about it’. This lack of education and understanding about mental health is arguably problematic as those with difficulties, whether diagnosed or undiagnosed, may find it difficult to seek help. It is well recognised that adolescents with mental health needs find seeking support difficult, in part due to stigma (Chandra & Minkovitz, 2006), and there is clearly limited transparency in discussions about these kinds of illnesses. However, teachers are on the ‘frontline’ and teachers in our study described how they tended to be a first choice for many seeking support, due to the long-standing relationships.

Yeah, because that’s why they confide in you as teachers because they see you every single day, they build that relationship over a year.

P3–London education

But the very nature of our jobs, the kids talk to us whether we want them to or not (laughter).

P1–London education

### Theme Four: The Responsibility of Parents for Their Children’s Mental Health

While there is a growing emphasis on schools to promote mental health, prevent mental illness and support those with diagnosed conditions, adolescents do not exist within an educational vacuum. Participants, in debating the extent of the role of teachers, referred to the broader responsibility outside of school, and specifically negotiated the role parents play in supporting mental health.

I think if their parents are supportive it makes a massive difference, so us being supportive in school, but if they’ve not got support at home as well, then let’s face it, it doesn’t matter how much we see them, the parents are the biggest influence in their lives

P2–Leicester education

But I think, I just don’t think and this is probably a judgemental perspective but I don’t think enough parents put the needs of children first

P1–CAMHS1

You’ve got that factor where parents can’t identify it and so they think it’s a problem for the school.

P8–Leicester education

Participants argued that parents play an important role in the mental well-being of their children. It was reported that when ‘parents are supportive’ there was a greater chance for those children having their needs met, with the rationale that ‘parents are the biggest influence in their lives’, but when ‘parents can’t identify it’ then parents position the problem as one for the school to deal with. They argued that despite teachers providing support, if parents fail to do so then this support will not be adequate. Indeed, some participants felt that parents did sometimes fail to provide the support that adolescents need, and that this created a difficulty for young people as their parents were not putting ‘the needs of children first’. Some extended this claim by arguing that teachers provided greater support than parents, and this may be the explanation for why adolescents sought help from school, rather than home.

I think maybe teachers on the whole are more sympathetic than parents are.

P3–London education

By positioning teachers as ‘more sympathetic than parents’, a certain dynamic between adolescents and education was constructed by participants. In so doing, they reported the reason adolescents sought help in the education environment reflected the personal styles of educationalists, with the implication that this is important for help-seeking and mental health. Indeed, it is important that adolescents seeking mental health support do not feel judged or embarrassed when confiding their difficulties (Chandra & Minkovitz, 2006). Nonetheless, parents play an essential role, and participants felt that parents themselves needed greater support and knowledge to equip them to support their children.

So maybe we should do more with parents to educate them, or to help them to help them to understand mental health issues and reduce stigma with parents.

P2–London education

I think it has a lot of stigma attached to it still in schools I don’t think it has been explored in enough detail to help people deal with certain things

P7–London education

They should have support in school and we need to do all the things we’ve talked about but I think it’s very, very important that parents have more understanding and the kids are able to tell them when they’re going through these things.

P1–Leicester education

Improving parents’ understanding of mental health is arguably a positive step forward, and participants argued that it is ‘very important that parents have more understanding’, but also that parents are open to hearing about their child’s problems. Stigma and mental health affects parents (Corrigan & Miller, 2004) and arguably educating them can help to ‘reduce stigma with parents’ as there is ‘a lot of stigma attached to it’ and in turn could reduce stigma for adolescents too. However, parents often need support in recognising their child needs mental health support, and in knowing how to respond in such cases, as well as where to go beyond the school for more specialist intervention. Participants argued that parents play an important role in keeping their children mentally healthy, but that they needed external help in developing those skills. Mental health practitioners particularly argued that reducing the likelihood for the need for specialist services relied heavily on parental intervention.

And so now you have less social support for parents who are struggling to parent their kids and keep them healthy.

P1– CAMHS 1

Building up those skills in the parents and carers so that they don’t end up in the situation where people are working with them.

P3–CAMHS 2

Mental health practitioners recognised that parents in a contemporary society have ‘less social support’ and may need support in ‘building up those skills’. Mental health was argued to be complex and because of the growing prevalence it was claimed that parents were central to mental health promotion and prevention, but needed help in achieving that.

## Discussion

There is a growing government directive to tackle mental health in schools, which is a global issue. Juxtaposed with higher academic targets in education (HCEHC, 2017), building resilience, promoting well-being, and preventing mental disorders is high on the educational agenda for many countries, and particularly the UK. Training teachers in mental health and resilience is viewed as central to tackling the rising prevalence of childhood disorders (PHE, 2017), and yet, arguably, this fails to demonstrate an understanding of the complexity of mental health difficulties, the challenges of meeting need, quality of training and the perspectives of key stakeholders. While there is clearly a role for schools in promoting well-being, reducing stigma and teaching adolescents about mental health issues, the extent to which this can be integrated into the educational role, and the reach of this is more questionable.

We argue that meeting mental health needs should be an important focus for education, but that this needs to be integrated within a ‘whole school approach’ to account for the various layers woven into the education system (Weare, 2000); an approach that takes a multi-tiered approach to mental health, meaning it incorporates all school personnel and interacting with external agencies for promotion, prevention and intervention (Vaillancourt, Cowan, & Skalsk, 2016). Indeed, partnerships between schools and CAMHS can be central to identifying and supporting unmet need, as well as educating those who work in and with schools (Svirydzenka, Aitken, & Dogra, 2016). Schools have an important role to play within the broader mental health rubric, as attending to well-being facilitates positive outcomes, including academic attainment (DfE, 2014). However, it is important that any role that schools play in this is well resourced, providing the necessary knowledge and skills to teachers, as well as support for their own mental health, and recognises the limits to this expectation.

Notably, there is only a limited evidence base exploring the feelings and ideas of frontline professionals, and adolescents, on the matter of teachers’ role in mental health in schools, and in this article, we have sought to address that. While our original focus was on social media and mental health promotion, it is testimony to the real-world concerns in schools that participants in all groups veered away from that during discussions, and oriented to the broader issues of mental health responsibility in schools. This signifies the emphasis of concern related to the readiness of frontline educators to play a role and the parameters of that role. Evidently, mental health responsibility in schools is a central concern for those on the frontline, and the views of educationalists, adolescents and mental health practitioners seem to reflect the larger national policy concerns and the strong feelings and ideas around these issues were prominent despite a slightly different overall focus for the project.

The remit and responsibility of schools was viewed as important by all participants. Educating adolescents about mental health and having conversations that were educational were argued to be within the remit of schools, and adolescents specifically claimed that they relied on teachers for some support and education about such issues. Nonetheless, generally, participants reported that teachers lacked the skills or knowledge to adequately address mental health in schools. A lack of time, training and resources, as well as limited opportunity for debriefing or supervision, were reported to be the primary challenges facing teachers in relation to growing expectations and pressures on their role. Indeed, our research participants felt that the lack of opportunity to debrief and offload the emotional impact of dealing with troubled adolescents had a negative impact on their own mental health. This is especially troubling as a recent survey conducted by the National Union of Teachers found that nearly 50% of the 3000 teachers surveyed were planning to leave the field, with one prominent reason being their own mental well-being (The Guardian, 2017).

Of interest in this article was the conflation between different levels of mental health issues. Participants fluidly moved from talking about general educational issues of helping adolescents to be more mental health aware, promoting their mental well-being and reducing stigma through conversation, to more severe issues such as supporting those diagnosed with severe mental illness, suicidal ideation and self-harm, and emotional trauma. Thus, a multi-tiered approach focusing on mental health promotion, prevention of mental illness and interventions for those diagnosed (Vaillancourt et al., 2016) within a whole school framework is important (Weare, 2000). The roles and responsibilities of schools in dealing with mental health must account for the multidimensional and complex issues related to mental health and mental health difficulties, and the great diversity of needs that will be encountered within the school environment. Arguably, teachers with a greater knowledge and understanding of mental health will be better equipped at all levels, and yet, they are not currently well resourced or supported to manage the increasing prevalence of problems.

There is some optimism that mental health will receive more support in the future. Since June 2017, funding has been secured to provide secondary (high) schools mental health training. By 2020, the aim is for all secondary schools to have a member of staff identified as a ‘mental health champion’ with responsibility to support well-being and promote resilience (PHE, 2017). Indeed, the current Secretary of State for Health, Jeremy Hunt (PHE, 2017), is reported as claiming that ‘identifying symptoms of mental illness early can help young people on the road to recovery’ (n.p). While training teachers to identify mental health difficulties is important and ultimately could benefit adolescents and their families, in isolation it will be insufficient. Arguably, any training provided for teachers should not just focus on the promotion of mental health, but also the practicalities of strategies to support in-class teaching of adolescents with mental health difficulties (Rothi et al., 2008). The promotion of a whole school approach by DoH and DfE (2017) advocates schools should tackle mental health and well-being through their behaviour policy, curriculum design, care and support for pupils and staff, and engagement of parents. While this seems a sensible approach, there is limited research evidence to support schools in identifying best practice.

Thus, while the commitment of new funding for training teachers is a positive beginning to addressing a large-scale problem, it should not be viewed as the solution, but as part of an ongoing process which sees greater funding levied at mental health (Karim & O’Reilly, 2017). Indeed, Sims-Schouten (2017) argued that this funding metaphorically represents merely a ‘sticking plaster solution’ to the challenge that child and adolescent mental health brings society (n.p). Sims-Schouten noted that a 2-day course with limited funds was insufficient to cover the huge diversity of age and gender differences in mental health, and thus, any impact is likely to be limited. Furthermore, it will not account for the role of parents and community in both contributing to and assuaging emotional distress, which as we have demonstrated through the perspectives of key stakeholders is central in dealing with the scale of the problem. Clearly, there are concerns about managing the increasing responsibility placed on educators and the role that teachers play in promoting, preventing and managing mental health, especially in terms of funding, training and confidence. These were core issues raised as central by participants, despite these issues not reflecting the research agenda.

This is because more broadly child and adolescent mental health has not received the attention it deserves. Families often do not receive appropriate levels of support until the child is at crisis point (DoH, 2015). In 2004, only 25% of children were receiving appropriate treatment for their mental health need (Green et al., 2005); this remained unchanged by 2010 (LSEPS, 2012). This is due to deeply concerning difficulties with funding and delivery of mental health services (HCHC, 2014). Thus, a fundamental problem for teachers to signpost to other services when recognising a need beyond pastoral care (emotional support and promotion of well-being) in the educational environment. Schools also need support in how best to cater for an adolescent where it is acknowledged that specialist input is required, but there is often significant delay in access to this.

The rhetoric that mental health is a priority is not being fully realised with commitments of funding. Any management of mental health responsibility needs to be carefully navigated, in consultation with those in education and mental health services, listening to what adolescents want from them. Adolescents’ mental well-being and mental health is a primary concern for parents (Burns, 2015); yet, the funding for mental health remains woefully inadequate, putting a great strain on children’s services. Lack of funding has consistently remained the significant barrier for mental health support for adolescents, with local commissioners typically failing to account for how and where any money is spent (Sims-Schouten, 2017). Emphasising responsibility for mental health is likely to be resisted by those on the frontline, risk teachers’ personal mental well-being, and may even reduce the number of teachers in the profession.

Mental health and the responsibility of schools is an area of concern that requires a great deal more research attention. The voices of children and adolescents across the age spectrum are crucial in informing policy decisions and ascertaining areas of priority. Equally, those working in schools and in mental health services play a central role in delivering support and information to young people and their voices need to be represented in decisions made about future directions. The participants in our sample reflected considerable diverse ethnic populations and thus demonstrates that the concerns raised in this paper reflect broad cultural perspectives. However, the unique perspectives of different ethnic groups do require a more in-depth examination to better understand the diversity of need in schools.

In conclusion, although teachers may be able to support well-being, promote mental health and resilience in adolescents, they are not in a position to treat mental illness, but will nonetheless be working with families and signposting to other areas of support. It is possible that upskilling teachers to recognise issues may result in an increase in referrals to specialist services, as they try to find ways of signposting concerned parents beyond the school. However, if access to these services remains limited there is a risk of greater pressure on teachers, and greater problems for adolescents and their families.

## Footnotes

1 The Key is an organisation who provide information to the education sector, to school leaders and governors.

2 PSHE is a class taken by many in England, as part of a programme to equip adolescents with an understanding of risk. It is a school subject through which pupils develop the knowledge, skills and attributes they need to keep themselves healthy and safe, and prepare for life and work in modern Britain.

3 We recognise that not all mental health practitioners are medically trained either, but the context of the contribution (which cannot all be shown here for space reasons), was that teachers do not have specialist mental health training or skills in the same way that clearly mental health practitioners take years in acquiring.
